# Long‐Term Stability of Ethyl Glucuronide in Hair: A 10‐Year Retrospective Analysis of 909 Samples by LC–MS/MS

**DOI:** 10.1002/dta.3934

**Published:** 2025-07-22

**Authors:** Sara Casati, Alessandro Ravelli, Roberta F. Bergamaschi, Massimo Del Fabbro, Giorgio Binelli, Gabriella Roda, Marica Orioli

**Affiliations:** ^1^ Laboratorio di Tossicologia Forense, Dipartimento di Scienze Biomediche, Chirurgiche ed Odontoiatriche Università degli Studi di Milano Milan Italy; ^2^ Fondazione IRCCS Cà Granda Ospedale Maggiore Policlinico Milan Italy; ^3^ Dipartimento di Scienze Biomediche, Chirurgiche ed Odontoiatriche Università degli Studi di Milano Milan Italy; ^4^ Dipartimento di Biotecnologie e Scienze della Vita Università dell'Insubria Varese Italy; ^5^ Dipartimento di Scienze Farmaceutiche Università Degli Studi di Milano Milan Italy

**Keywords:** alcohol biomarkers, ethyl glucuronide, forensic toxicology, hair analysis, long‐term stability

## Abstract

Monitoring long‐term alcohol consumption is critical in forensic and public health contexts. Hair analysis of ethyl glucuronide (EtG), a direct metabolite of ethanol, has become a standard method for detecting chronic alcohol use. While the reliability of EtG hair testing is well established for short‐ and medium‐term analyses, its stability in hair stored over extended periods has not been comprehensively evaluated. This limitation is especially relevant in retrospective investigations, postmortem evaluations, and long‐term epidemiological studies, where archived samples may be analyzed years after collection. In this study, we assessed the long‐term stability of EtG in human hair stored for up to 10 years. A total of 909 samples originally analyzed between 2013 and 2022 were re‐tested in 2023 using a previously published and validated liquid chromatography–tandem mass spectrometry (LC–MS/MS) method. When the results of the old and the new analyses were compared, EtG concentrations showed no significant degradation over time, with more than 80% of the samples displaying matching values when analytical uncertainty was considered. Only a small fraction of samples (4.4%) dropped below the commonly used interpretive threshold for chronic alcohol use (30 pg/mg) after 10 years of storage. These findings provide robust evidence that EtG remains chemically stable in hair under standard storage conditions over a decade, confirming the reliability of archived samples for assessing alcohol use history and expanding the utility of EtG analysis in long‐term toxicological and forensic investigations. The demonstrated stability strengthens confidence in hair as a matrix for retrospective substance use evaluation across scientific disciplines.

## Graphical abstract

EtG hair analysis has a standard method for detecting alcohol abuse. In this study, we assessed the long‐term stability of EtG in hair stored up to 10 years. A total of 909 samples originally analyzed between 2013 and 2022 were re‐tested in 2023 by LC‐MS/MS. Etg showed no significant degradation over time, with more than 80% of the samples dislaying matching values. These findings proved robust evidence that EtG remains chemically stable in hair under standard storage conditions over a decade.

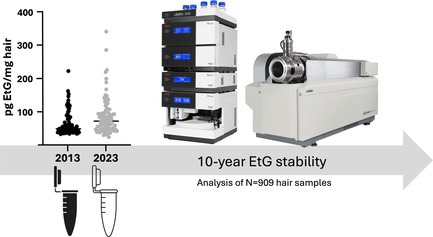

## 1 Introduction

The detection of chronic alcohol consumption is essential for both forensic and clinical purposes. This information may be necessary for legal actions such as those involving drunk driving on public or private transportation, assessing eligibility for social health care, or resolving family matters like child custody. Additionally, the evaluation of chronic alcohol abuse plays a crucial role in decisions about liver transplant eligibility, screening for alcohol use disorders (AUDs), monitoring relapse, postmortem investigations, and workplace testing. Therefore, the development and availability of reliable diagnostic tools are of paramount importance.

Among these tests, the quantification of ethyl glucuronide (EtG) in hair has emerged over the past 10–15 years as the most reliable marker in both forensic and clinical studies [1, 2, 3]. In the past few years, another EtG analysis application emerged relative to epidemiologic studies, to better define the correlation between alcohol consumption and some chronic diseases and mortality. EtG is a non‐oxidative, volatile, hydrophilic, and stable phase II ethanol metabolite. It constitutes an ethanol metabolite: only approximately 0.1% to 0.2% of ingested ethanol is converted to EtG, resulting from glucuronidation by UDP‐glucuronosyltransferase (UGT), which adds a glucuronic acid moiety to the ethanol molecule, primarily in the liver, although glucuronosyltransferase activities are also present in the kidneys and intestines [4, 5]. EtG can be quantified in different biological matrices, reflecting alcohol consumption over different time frames. In keratin matrices such as nails and hair, EtG values provide evidence of alcohol use over several months, depending on the length of the sample [2, 3].

Understanding the stability of EtG in hair samples is critical for confirming in scenarios that may require analysis years after sample collection. For example, in postmortem cases, determining alcohol consumption history can be useful for establishing the cause of death. Similarly, archived samples may be revisited in family law cases or in long‐term epidemiological studies. Moreover, long‐stored hair samples may be used for epidemiological research studies, and their reliability in terms of EtG content is pivotal.

To date, no studies have been designed yet to investigate changes in EtG levels in hair after such long storage periods. Previous research has demonstrated EtG stability in hair for a few months. In particular, Agius et al. [6] in 2012 showed that EtG can be reliably determined in hair samples up to 12 cm long (where each cm of hair corresponds to approximately 1 month). Other studies focused their attention on aspects such as the best hair segment to analyze [7] or the influence of permanent coloring and bleaching on the interpretation of EtG concentrations [8, 9]. Moreover, EtG stability was investigated in hair submitted to accelerated aging between 4°C and 60°C for periods between one and 24 months [10].

Therefore, the aim of this study was to assess EtG stability in hair samples stored for up to 10 years using LC–MS/MS analysis. Establishing this long‐term stability is pivotal to confirming the utility of hair EtG analysis in retrospective assessments of alcohol use.

## 2 Materials and Methods

### 2.1 Chemicals and Reagents

EtG and EtG‐*d*_5_ (internal standard [IS]) were purchased from Sigma Aldrich (Milan, Italy) as methanolic solutions (100 μg/mL). Ultrapure water (H_2_O), methanol (MeOH), dichloromethane (DCM), acetonitrile (ACN) and formic acid (98%) were provided by Sigma Aldrich (Milan, Italy). The ultrapure water and solvents were of suitable grade for high‐performance liquid chromatography–mass spectrometry (HPLC‐MS).

### 2.2 Hair Samples

#### 2.2.1 Collection of Hair

Scalp hair was collected (*N* = 909) by cutting hair close to the skin in the occipital or vertex region of the head. Only the proximal ≤ 6 cm segment was collected from individuals, tested in our laboratory for alcohol abuse over a 10‐year period (2013–2022), and kept at room temperature (approximately 20°C–25°C) and away from light. Evaluation for alcohol abuse was requested for driving license renewal. All the subjects involved in the study gave their written informed consent for the anonymous use of their samples for research purposes.

#### 2.2.2 EtG‐Free Hair

EtG‐free hair of adult teetotalers (*N* = 10) was collected, analyzed to avoid any chromatographic interferences, and mixed to generate a uniform sample of blank hair for EtG calibrators and quality controls.

### 2.3 Hair Sample Extraction and HPLC‐MS/MS Analysis

EtG hair extraction and analysis were performed according to a previously published and accredited to DIN EN ISO/IEC 17025 standards LC–MS/MS in‐house method [9]. Any changes in instrumental and method validation during the 10‐year period were reported in Table S1. In brief, 4‐mL DCM and 2‐mL MeOH were used to wash the samples, which were dried and scissor‐cut into 2–4 mm pieces; an aliquot (20–120 mg) from each sample was placed into 2 mL Eppendorf tubes and added with 900 μL of H_2_O/MeOH (88:2, v/v) and 100 μL of EtG‐*d*_5_ (100 ng/mL in H_2_O). After overnight incubation and extraction at room temperature, an extra ultrasonication of 2 h was performed. One hundred microliters of the supernatant was transferred into an autosampler vial for the analysis. Analyses were performed on a Dionex UltiMate 3000 HPLC system (Thermo Fisher Scientific, Waltham, MA, USA) coupled to a QTRAP 4000 triple quadrupole mass spectrometer (Sciex, Darmstadt, Germany) operating in negative electrospray ionization (ESI) mode. Compounds were separated on a Luna Omega Polar C18 column (100‐mm length × 2.1‐mm i.d, 3 μm particle size) fitted with Polar C18 (4 × 2.0 mm) upstream pre‐column (Phenomenex, CA, USA) at 25°C using 0.1% formic acid in H_2_O (A) and 0.1% formic acid in acetonitrile (ACN) (B) as mobile phases, with the following 10‐min linear gradient: 0 to 2 min (2.5% B), 2 to 3 min (20% B), 3 to 4.5 min (60% B), 4.5 to 5.5 min (linear increase to 100% B), 5.5 to 7 min (100% B), 7 to 7.1 min (decrease to 2.5% B) and 7 to 10 min (reconditioning 2.5% B). The flow rate was set at 0.4 mL/min. The limits of detection (LOD) and of quantification (LOQ) were 2 and 6.7 pg/mg for EtG. The coefficient of correlation (R) was consistently better than 0.99 when spiked to EtG‐free hair samples and extracted. Inter‐run repeatability determined by the relative standard deviation (RSD) was overall < 15%.

The analytical method, accredited to DIN EN ISO/IEC 17025 standards, and the instrumentation (Dionex UltiMate 3000 HPLC system coupled to a QTRAP 4000 triple quadrupole mass spectrometer) were consistently used without significant modifications throughout the entire 10‐year study period, except for early 2013, as reported in Table S1. Limits of detection (LOD) and quantification (LOQ) remained stable at 2 and 6.7 pg/mg, respectively. Routine maintenance and replacement of consumables were performed under our accredited quality system, ensuring method continuity and performance consistency. The laboratory also participated successfully and continuously in external proficiency testing (PT) schemes for EtG in hair during the 10 years, with results consistently falling within acceptable ranges, thereby confirming the reliability and comparability of the original and repeat analyses.

### 2.4 Study Design

Nine hundred and nine randomly selected hair samples that had been originally tested for EtG (T0 result >/= 15 pg/mg), on a variety of dates between 2013 and 2022, at the Forensic Toxicology Laboratory of the University of Milan, were retested in April 2023. Originally, all samples were split into two locks before analysis: one lock was segmented according to the clients' request and analyzed as described above (first test). The remaining intact hair lock was wrapped in a paper foil, enclosed in a sealed plastic envelope, and stored at room temperature in dry and dark conditions for a period between 1 and 10 years before being retested at the same length as the first one (second test). The results were categorized into 11 groups based on the length of time between the first test and the second test. Data are presented as mean ± SD with median and range. In this study, a threshold value of 30 pg of EtG per milligram of hair was established as the cut‐off point for distinguishing between negative and positive cases of alcohol consumption [11].

### 2.5 Statistical Analysis

Statistical analyses were performed using GraphPad Prism 7 (GraphPad Software, CA, USA) and in the R environment (R Core Team, 2018). The total *N* = 909 samples were divided into two tests: the first test at the sampling data (between 2013 and 2022) and the second test (re‐analysis) in 2023. Data for the two test points (first and second tests) are presented as scatter plots. The horizontal line represents the median value. The EtG concentration was tested among each of the two test points by a paired Student's t‐test (t.test procedure of R, with Welch's adjustment for possible inequality of variances), after logarithmic transformation for normality. In all tests, the significance level was set at *p* < 0.01. To avoid increasing the chance of Type I error due to multiple comparisons, a Benjamini–Hochberg test was run to adjust the *p*‐values of the conducted *t* tests [12].

To assess the stability of EtG concentrations over long‐term storage, we considered a sample stable if the difference between the first and second test results was within the analytical measurement uncertainty calculated for each individual result (y = 0.1732 × EtG + 0.1157). This approach aligns with the concept of critical difference described by Stamm [13] as it defines the smallest change that can be interpreted as a true difference beyond analytical imprecision. This criterion is concentration‐dependent, applying consistently across the EtG measured range (15–400 pg/mg), with non‐significant degradation defined by differences within the expected imprecision at each concentration level.

## 3 Results

EtG concentration values, expressed as mean value ± SD, determined in the first and second tests (2023), are presented in Table 1. By ‘first test’ we refer to hair sample analyses conducted between 2013 and 2022, whose values are then compared with those from the ‘second test’, carried out in 2023. Individual values are not shown due to their large number, about *N* = 100 samples for each year, except for 2020 (*N* = 40).

**TABLE 1:** EtG concentration in the first test and in the second test (2023): mean value ± SD (≤ 6 cm proximal hair sample).

Year of sampling	First test (pg/mg hair)	Second test (pg/mg hair)	*p*	*p* after BH correction
	Mean ± SD			
2013	63.79 ± 35.64	85.23 ± 54.52	0.001227*	0.006135*
2014	80.87 ± 79.85	72.57 ± 65.66	0.02033	0.0407
2015	71.76 ± 67.82	70.03 ± 63.21	0.1967	0.2459
2016	77.42 ± 41.49	77.44 ± 45.54	0.8842	0.8842
2017	75.48 ± 48.96	72.85 ± 48.51	0.03385	0.0846
2018	75.79 ± 49.20	87.02 ± 53.45	0.03326	0.0554
2019	87.38 ± 62.86	105.30 ± 87.73	0.002135*	0.0071*
2020	90.94 ± 73.05	98.07 ± 69.69	0.0494	0.0706
2021	75.93 ± 46.65	81.29 ± 59.08	0.3773	0.4192
2022	47.30 ± 43.15	40.38 ± 39.14	3.1 × 10^−6^ *	3.1 × 10^−6^ *

Abbreviation: BH = Benjamini–Hochberg.

* *p* < 0.01 (first vs. second test points, Student's *t* test).

To further confirm the reliability of the analytical method and the consistency of laboratory performance over the 10‐year period, we evaluated the results of external proficiency tests (PTs) conducted during the same timeframe. The outcomes of these PTs provide an independent measure of the laboratory's analytical accuracy and support the validity of the EtG concentration data presented in this study.

**FIGURE 1:**
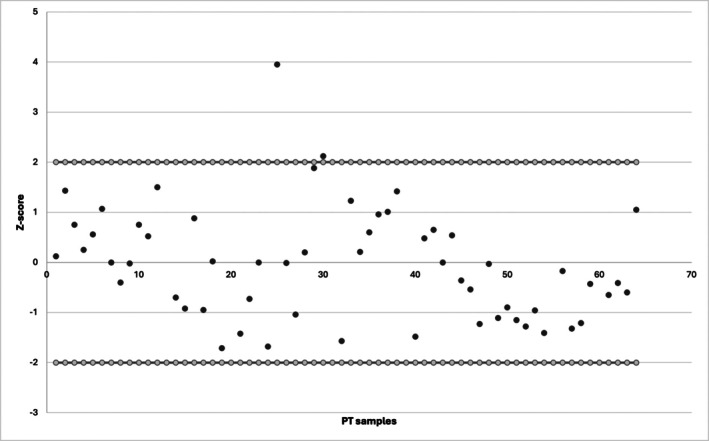
Summary of the *Z*‐scores of 64 external proficiency tests (PT) performed by our laboratory from 2013 to 2023 (red dots represented the *Z*‐score limits set at ±2).

Between 2013 and 2023, our laboratory participated in a total of 64 external proficiency tests (PTs) organized by GTFCH and CRCMED LAB, covering a wide range of EtG concentrations from below the limit of quantitation (LOQ) to over 80 pg/mg. Throughout this period, the majority of the *Z*‐scores obtained were well within the acceptable range of ±2, confirming the accuracy and reliability of our analytical performance over the 10‐year span. Specifically, only two PT results exceeded the ±2 threshold (*Z*‐score of +3.95 in 2017/01A and +2.12 in 2018/01B), corresponding to a deviation above the upper limit, while all other exercises produced *Z*‐scores indicative of satisfactory performance. In particular, several exercises showed *Z*‐scores close to zero, further demonstrating excellent agreement with the target values. The overall trend of *Z*‐scores did not display systematic drift over time, supporting the stability and comparability of our results across different PT providers and years. A graphical summary of the *Z*‐scores, including their distribution relative to the ±2 acceptance limits, is presented in Figure 1 and Table S1.

EtG concentration values were log‐transformed to normalize their distribution prior to statistical analysis. After transformation, a visual analysis of the quantile‐quantile plots (Q‐Q plots) was performed. Because of the very large sample size, a paired Student's t‐test was performed. The significance level was set at 0.01 due to the applied objectives of the analysis, prioritizing increased protection of the test over the risk of overestimating the significance of small effects.

Student's t‐test indicated that the second test of EtG was not significantly different from the first test at the *p* < 0.01 level, except for the years 2013, 2019, and 2022 (*p* = 0.0012, *p* = 0.0021, *p* = 3.11 × 10^−6^, respectively).

Analysis of the data set reveals that there is no downward trend for the EtG content in the samples tested (see Table 2, in which the median value and the range between minimum and maximum level are reported).

**TABLE 2:** EtG value in the first test and in the second test (2023), showing the median value as well as the minimum and maximum concentrations observed (≤ 6 cm proximal hair sample), and percentage of the mean EtG variation.

Year of sampling	First test (pg/mg hair)		Second test (pg/mg hair)		Mean EtG variation %
	Median	Max/min	Median	Max/min	
2013	48	222/33	70	340/24	83.1
2014	52	473/32	55	411/11	25.7
2015	50	443/15	53	408/9	30.4
2016	60	234/35	66	228/18	24.2
2017	58	329/36	57	231/LOQ	34.1
2018	57.5	328/15	70	308/10	35.3
2019	63	327/28	72	442/27	34.2
2020	66	412/15	76	390/LOQ	30.1
2021	64	380/15	69.5	414/LOQ	34.8
2022	28	257/18	28	244/LOQ	19.9

Abbreviation: LOQ = limit of quantitation.

Table 2 shows, for each couple of data (first vs. second test), the mean level variation in absolute terms F. The variation is higher for samples tested in 2013, but since 2014, the level variation seems to be almost stable.

The percentage of matching results, between the first test and the second test (2023), calculated considering the measurement uncertainty (first test in pg/mg of hair ± measurement uncertainty vs. second test in pg/mg of hair ± measurement uncertainty), is reported in Table 3. The measurement uncertainty (*y*) was calculated by the following equation *y* = 0.1732*x* + 0.1157, where *x* is the EtG concentration in pg/mg, and it was evaluated considering the relative contribution of working solutions (reference standard materials, dilutions), sample preparation (laboratory scale and micropipettes), calibration curve (BIAS), and repeatability (standard deviation), as previously published by Casati et al. [9]. A lower level of matching results is again observable in the set of samples dating from the year 2013, while they seem to be stable for all the other sets of samples (80.2 ± 5.7, mean ± SD).

**TABLE 3:** Number of cases per year and percentage (%) of matching EtG value (≤ 6 cm sample) between the first and the second tests (2023).

Year of sampling	Number of cases	% Matching samples
2013	97	67.4
2014	96	77.7
2015	96	80.4
2016	40	76.9
2017	100	78.3
2018	100	75.0
2019	100	89.9
2020	99	84.8
2021	99	72.4
2022	97	86.6

## 4 Discussion

This study provides new and robust evidence supporting the long‐term stability of EtG in human hair, a key biomarker for monitoring chronic alcohol consumption. Our findings indicate that EtG concentrations remain largely stable over a storage period of up to 10 years at room temperature and in the dark. This has substantial implications for the forensic and clinical utility of archived hair samples in retrospective evaluations of alcohol use.

Furthermore, by comparing the differences between the first and second test results with the analytical measurement uncertainty for each sample, we effectively applied a stability criterion equivalent to the critical difference approach [13]. This allowed us to interpret the absence of significant EtG degradation over time, confirming the long‐term stability of EtG in hair within the expected analytical imprecision.

Although previous research has demonstrated the short‐ to medium‐term stability of EtG in hair [8, 10], our work represents the first large‐scale, longitudinal analysis covering a full decade of storage.

Notably, we observed no consistent downward trend in EtG concentrations across the tested samples. In fact, in a substantial portion of cases, EtG values were either unchanged or slightly elevated during reanalysis. While slight fluctuations were observed—especially in the earliest group (2013)—these differences are likely attributable to factors unrelated to EtG degradation, including inter‐operator variability, minor inconsistencies in sample handling, or differences in the original hair lock's anatomical collection site. Additionally, long‐term storage could have determined a partial hair misalignment. Moreover, any changes in equipment could potentially affect EtG quantification; however, the laboratory consistently and successfully participated in external proficiency testing (PT) over the 10‐year period, as reported in Figure 1 and in Table S1. Among 64 PTs, only two exceeded the *Z*‐score threshold of ±2.

These results are in line with the work of Ammann et al. [10] who demonstrated that EtG remains stable under accelerated aging conditions and extend the findings of Crunelle et al. [8].

The high percentage of matching results between the two test points (mean 78.9% ± 6.8) further supports the reliability of archived hair samples for long‐term analysis, especially considering the estimated measurement uncertainty.

This trend is also consistent with preliminary results recently presented by Duda and Tsanaclis, who found no significant degradation of EtG in hair stored up to 36 months under dry, dark, room‐temperature conditions, although a decrease beyond that point was suggested. Our findings, covering a 10‐year span, extend this observation and support the long‐term reliability of EtG in hair well beyond the 3‐year threshold [14].

**FIGURE 2:**
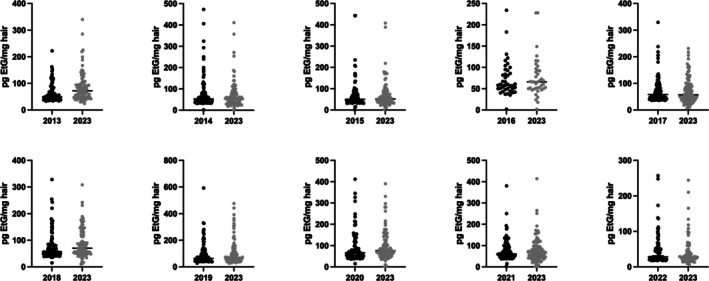
Scatter plots representing all the data for the two groups (first and second test). The horizontal line represents the median value.

As illustrated in Figure 2, the scatter plots of EtG concentrations from both the first and second analyses show a high degree of concordance, with median values (horizontal lines) remaining consistent across the two time points. This visual distribution supports the statistical finding of long‐term stability, as no systematic degradation trend is evident even after 10 years of storage.

Importantly, the potential rate of false negatives after 10 years was limited to 4.4% (*N* = 40 out of 909) when applying the commonly used 30 pg/mg cut‐off. This supports the interpretation of archived samples even in borderline or threshold‐sensitive forensic contexts. Our findings reinforce the stability profile of EtG in keratinized matrices, further consolidating its status as a reliable long‐term biomarker for alcohol intake [15, 16].

The variability in hair EtG concentrations observed in 2013 (Tables 1, 2, and 3) may be related to methodological variables, as reported in Table S1. However, no evidence of chemical degradation per se was observed. Given that hair is a biologically inert matrix with low enzymatic activity post‐collection, it is plausible that EtG, once incorporated, remains chemically stable for years—consistent with prior biochemical characterizations of glucuronide conjugates [3, 10].

Overall, the current study adds a critical time dimension to the existing literature, offering forensic and clinical professionals' greater confidence in the interpretation of delayed or archived hair samples.

## 5 Conclusions

Our results demonstrate that the concentration of EtG in hair remains stable for at least 10 years when stored at room temperature and protected from light. These findings validate the forensic reliability of long‐term archived hair samples and support the assumption that valid results of EtG in hair can be expected in retrospective toxicological investigations, including post‐mortem evaluations, historical alcohol consumption analysis, and long‐term epidemiological research. Further studies using other biomarkers and matrices may help generalize this approach to long‐term substance use assessment.

## Conflicts of Interest

The authors declare no conflicts of interest.

## Supporting information


**Table S1.** Scheme of external proficiency tests (PT) performed by our laboratory from 2013 to 2023, including the provider, target value, our laboratory's result, and the corresponding *Z*‐score (highlighted in bold where the test was not passed).

## Data Availability

The data that support the findings of this study are available from the corresponding author upon reasonable request.
