# Supplementary material for: Long‐Term Stability of Ethyl Glucuronide in Hair: A 10‐Year Retrospective Analysis of 909 Samples by LC–MS/MS
Source: Drug Test Anal. 2025 Jul 22;17(11):2239–45. doi: 10.1002/dta.3934 (PMC12580159; doi:10.1002/dta.3934)
Supplement: Supplementary file 1 — Table S1. Scheme of external proficiency tests (PT) performed by our laboratory from 2013 to 2023, including the provider, target value, our laboratory's result, and the corresponding Z‐score (highlighted in bold where the test was not passed). [file DTA-17-2239-s001.pdf]

**Table S1.** Scheme of external proficiency tests (PT) performed by our laboratory from 2013 to 2023, including the provider, target value, our laboratory’s result, and the corresponding Z-score (highlighted in bold where the test was not passed).

| Provider | Year/Exercise number | Sample | Target value pg/mg | Lab result pg/mg | Z-score | Z-score limits | |  |  |  |  |  |  |  |  |
| --- | --- | --- | --- | --- | --- | --- | --- | --- | --- | --- | --- | --- | --- | --- | --- |
|  |  |  |  |  |  |  |  | GTFCH | 2013/01 | A | 41.6 | 43.0*° | 0.12 | 2 | -2 |
| GTFCH | 2013/01 | B | 59.9 | 81.0*° | 1.43 | 2 | -2 |  |  |  |  |  |  |  |  |
| GTFCH | 2013/02 | A | 47.0 | 56.0* | 0.75 | 2 | -2 |  |  |  |  |  |  |  |  |
| GTFCH | 2013/02 | B | 37.5 | 40.0* | 0.25 | 2 | -2 |  |  |  |  |  |  |  |  |
| GTFCH | 2013/03 | A | 21.5 | 25.0* | 0.56 | 2 | -2 |  |  |  |  |  |  |  |  |
| GTFCH | 2013/03 | B | 31.0 | 40.0* | 1.07 | 2 | -2 |  |  |  |  |  |  |  |  |
| GTFCH | 2014/01 | A | 7.0 | 6.7* | 0.01 | 2 | -2 |  |  |  |  |  |  |  |  |
| GTFCH | 2014/01 | B | 35.0 | 35.0* | -0.40 | 2 | -2 |  |  |  |  |  |  |  |  |
| GTFCH | 2014/02 | A | 5.9 | 15.0* | -0.02 | 2 | -2 |  |  |  |  |  |  |  |  |
| GTFCH | 2014/02 | B | 16.4 | 42.0* | 0.75 | 2 | -2 |  |  |  |  |  |  |  |  |
| GTFCH | 2014/03 | A | 11.2 | 13.0* | 0.52 | 2 | -2 |  |  |  |  |  |  |  |  |
| GTFCH | 2014/03 | B | 26.0 | 37.0* | 1.50 | 2 | -2 |  |  |  |  |  |  |  |  |
| GTFCH | 2015/01 | A | 4.0 | <LOQ* | - | 2 | -2 |  |  |  |  |  |  |  |  |
| GTFCH | 2015/01 | B | 11.9 | 11.9* | -0.7 | 2 | -2 |  |  |  |  |  |  |  |  |
| GTFCH | 2015/02 | A | 23.1 | 17.0* | -0.92 | 2 | -2 |  |  |  |  |  |  |  |  |
| GTFCH | 2015/02 | B | 44.0 | 54.0* | 0.88 | 2 | -2 |  |  |  |  |  |  |  |  |
| GTFCH | 2015/03 | A | 24.6 | 18.0* | -0.95 | 2 | -2 |  |  |  |  |  |  |  |  |
| GTFCH | 2015/03 | B | 23.8 | 24.0* | 0.02 | 2 | -2 |  |  |  |  |  |  |  |  |
| GTFCH | 2016/01 | A | 39.8 | 22.0* | -1.71 | 2 | -2 |  |  |  |  |  |  |  |  |
| GTFCH | 2016/01 | B | <LOQ | <LOQ* | - | 2 | -2 |  |  |  |  |  |  |  |  |
| GTFCH | 2016/02 | A | 21.1 | 9.0* | -1.42 | 2 | -2 |  |  |  |  |  |  |  |  |
| GTFCH | 2016/02 | B | 48.8 | 38,0* | -0.73 | 2 | -2 |  |  |  |  |  |  |  |  |
| GTFCH | 2016/03 | A | 1.2 | <LOQ* | - | 2 | -2 |  |  |  |  |  |  |  |  |
| GTFCH | 2016/03 | B | 59.6 | 35.0* | -1.68 | 2 | -2 |  |  |  |  |  |  |  |  |
| GTFCH | 2017/01 | A | 21.5 | 46.0* | **3.95** | 2 | -2 |  |  |  |  |  |  |  |  |
| GTFCH | 2017/01 | B | 32.1 | 32.0* | -0.01 | 2 | -2 |  |  |  |  |  |  |  |  |
| GTFCH | 2017/03 | A | 38.7 | 28.0* | -1.04 | 2 | -2 |  |  |  |  |  |  |  |  |
| GTFCH | 2017/03 | B | 53.3 | 56.0* | 0.20 | 2 | -2 |  |  |  |  |  |  |  |  |
| GTFCH | 2018/01 | A | 18.0 | 28.0* | 1.88 | 2 | -2 |  |  |  |  |  |  |  |  |
| GTFCH | 2018/01 | B | 27.4 | 44.0* | **2.12** | 2 | -2 |  |  |  |  |  |  |  |  |
| GTFCH | 2018/02 | A | 8.1 | <LOQ* | - | 2 | -2 |  |  |  |  |  |  |  |  |
| GTFCH | 2018/02 | B | 28.3 | 16.0* | -1.57 | 2 | -2 |  |  |  |  |  |  |  |  |
| GTFCH | 2018/03 | A | 31.5 | 42.0* | 1.23 | 2 | -2 |  |  |  |  |  |  |  |  |
| GTFCH | 2018/03 | B | 54.1 | 57.0* | 0.21 | 2 | -2 |  |  |  |  |  |  |  |  |
| GTFCH | 2019/01 | A | 16.1 | 19.0* | 0.60 | 2 | -2 |  |  |  |  |  |  |  |  |
| GTFCH | 2019/01 | B | 49.9 | 62.0* | 0.96 | 2 | -2 |  |  |  |  |  |  |  |  |
| GTFCH | 2019/02 | A | 27.3 | 35.0* | 1.01 | 2 | -2 |  |  |  |  |  |  |  |  |
| GTFCH | 2019/02 | B | 36.3 | 50.0* | 142 | 2 | -2 |  |  |  |  |  |  |  |  |
| GTFCH | 2019/03 | A | 5.7 | < LOQ* | - | 2 | -2 |  |  |  |  |  |  |  |  |
| GTFCH | 2019/03 | B | 22.5 | 13.0* | -1.48 | 2 | -2 |  |  |  |  |  |  |  |  |
| GTFCH | 2020/02 | A | 29.7 | 33.6 | 0.48 | 2 | -2 |  |  |  |  |  |  |  |  |
| GTFCH | 2020/02 | B | 44.3 | 51.8 | 0.65 | 2 | -2 |  |  |  |  |  |  |  |  |
| GTFCH | 2020/03 | A | 18.4 | 18.0 | 0.03 | 2 | -2 |  |  |  |  |  |  |  |  |
| GTFCH | 2021/03 | B | 26.9 | 31.0 | 0.54 | 2 | -2 |  |  |  |  |  |  |  |  |
| GTFCH | 2021/02 | A | 7.8 | 7.00 | -0.36 | 2 | -2 |  |  |  |  |  |  |  |  |
| GTFCH | 2021/02 | B | 25.9 | 22.0 | -0.54 | 2 | -2 |  |  |  |  |  |  |  |  |
| GTFCH | 2021/03 | A | 18.8 | 12.0 | -1.23 | 2 | -2 |  |  |  |  |  |  |  |  |
| GTFCH | 2021/03 | B | 35.7 | 36.0 | -0.03 | 2 | -2 |  |  |  |  |  |  |  |  |
| GTFCH | 2022/01 | A | 25.7 | 17.7 | -1.11 | 2 | -2 |  |  |  |  |  |  |  |  |
| GTFCH | 2022/01 | B | 52.2 | 40.3 | -0.90 | 2 | -2 |  |  |  |  |  |  |  |  |
| GTFCH | 2022/03 | A | 22.6 | 15.2 | -1.15 | 2 | -2 |  |  |  |  |  |  |  |  |
| GTFCH | 2022/03 | B | 47.5 | 31.9 | -1.28 | 2 | -2 |  |  |  |  |  |  |  |  |
| CRCMED LAB | 2022/01 | A | 35.4 | 25.9 | -0.96 | 2 | -2 |  |  |  |  |  |  |  |  |
| CRCMED LAB | 2022/02 | A | 22.1 | 16.3 | -1.41 | 2 | -2 |  |  |  |  |  |  |  |  |
| CRCMED LAB | 2022/03 | A | < LOQ | < LOQ | - | 2 | -2 |  |  |  |  |  |  |  |  |
| CRCMED LAB | 2022/04 | A | 12.5 | 12.0 | -0.17 | 2 | -2 |  |  |  |  |  |  |  |  |
| CRCMED LAB | 2022/05 | A | 28.9 | 15.8 | -1.32 | 2 | -2 |  |  |  |  |  |  |  |  |
| CRCMED LAB | 2022/06 | A | 20.0 | 11.0 | -1.21 | 2 | -2 |  |  |  |  |  |  |  |  |
| CRCMED LAB | 2023/01 | A | 13.7 | 15.5 | -0.43 | 2 | -2 |  |  |  |  |  |  |  |  |
| CRCMED LAB | 2023/02 | A | < LOQ | < LOQ | - | 2 | -2 |  |  |  |  |  |  |  |  |
| CRCMED LAB | 2023/03 | A | 22.0 | 25.5 | -0.65 | 2 | -2 |  |  |  |  |  |  |  |  |
| CRCMED LAB | 2023/04 | A | 12.0 | 12.9 | -0.41 | 2 | -2 |  |  |  |  |  |  |  |  |
| CRCMED LAB | 2023/05 | A | 41.0 | 46.3 | -0.60 | 2 | -2 |  |  |  |  |  |  |  |  |
| CRCMED LAB | 2023/06 | A | 36.0 | 25.4 | 1.05 | 2 | -2 |  |  |  |  |  |  |  |  |

* From 2013 to 2019, compounds were separated on a Gemini C18 column (150 mm length × 2.0 mm i.d., 3 µm particle size) with an upstream pre-column (Phenomenex, CA, USA) at 20 °C, using 0.1% formic acid and 10 mM ammonium formate (NH₄HCO₂) in H₂O (A) and acetonitrile (ACN) (B) as mobile phases, with the following 10-minute linear gradient: 0–2 min (5% B), 2–4 min (50% B), 4–4.5 min (linear increase to 95% B), 4.5–5.8 min (95% B), 5.8–6 min (decrease to 5% B), and 6–10 min (reconditioning at 5% B). Other equipment, detection limits, and sample preparation procedures did not change. The method was fully validated, and all acceptance criteria were satisfied and comparable to those of the current method.

° Analyses were performed on a QTRAP 3200 triple quadrupole mass spectrometer (Sciex, Darmstadt, Germany).

**Abbreviations:**

GTFCH = Gesellschaft Für Toxikologische Und Forensische Chemie (Society of Toxicological and Forensic Chemistry)

CRCMED = Centro Regionale di Coordinamento della Medicina di Laboratorio di Regione Lombardia
